# Attitudes of psychiatric staff toward coercion: nationwide AttCo study

**DOI:** 10.1192/bjo.2025.10808

**Published:** 2025-09-05

**Authors:** Klara Czernin, Anna Oster, Matthias Jaeger, Julia Junghanss, Josef S. Baumgartner, Lieselotte Mahler

**Affiliations:** Department of Child and Adolescent Psychiatry, Medical University of Vienna, Vienna, Austria; Department of Psychiatry, Charité University Medicine Berlin, Berlin, Germany; Department of Psychiatry and Psychotherapy, Clinics in the Theodor-Wenzel-Werk, Berlin, Germany; Psychiatrie Baselland, Liestal, Switzerland; Department of Psychiatry and Psychotherapy, Division of Social Psychiatry, Medical University of Vienna, Vienna, Austria

**Keywords:** Staff attitudes, coercion, ethics, mental health services, human rights

## Abstract

**Background:**

Attitudes of mental health professionals toward coercion are a potential tool in reducing the use of coercive measures in psychiatry.

**Aims:**

This study, part of the nationwide Attitudes toward Coercion (AttCo) project, aimed to assess staff attitudes on a nationwide and multiprofessional scale across adult, child and adolescent, and forensic psychiatric departments.

**Method:**

During 9 weeks in 2023, 1702 psychiatric staff members across Germany filled out a survey including gender, age, profession, work experience and setting, and the validated Staff Attitude to Coercion Scale (SACS). Analyses of variance and multivariate regression analysis for SACS mean overall score were computed to assess group differences.

**Results:**

Participants largely supported that coercion could be reduced with more time and personal contact (mean 4.20, range 1–5), and that coercion can harm the therapeutic relationship (mean 4.08); however, they acknowledged that coercion sometimes needs to be used for security reasons (mean 4.10). Regarding group differences, specialisation (*P* < 0.001) and professional affiliation (*P* = 0.008) remained significantly associated with SACS mean score (with a higher score in forensic psychiatric staff compared with staff in adult and child and adolescent psychiatry), when controlling for gender, age and work experience.

**Conclusions:**

Differences in attitudes are predominantly linked to professional training and structural surroundings. Professionals in adult psychiatry and child and adolescent psychiatry are more critical than staff in forensic settings, with an emphasis on patients’ rights and individuals’ integrity. Further studies are needed on how mental health professionals view coercion, and how actual use of coercion is influenced by staff attitudes.

The use and effects of coercive measures in the context of psychiatric treatment have been critically scrutinised and widely discussed since the introduction of the United Nations Convention on the Rights of People with Disabilities (CRPD).^1^ The controversial discourse concerns aspects such as care, safety and security, restriction of autonomy, violation of human rights and the use of force. Psychiatric staff are regularly confronted with this area of tension as they are obliged to make decisions and act accordingly. Their way of dealing with this is shaped by structural circumstances and individual factors such as personality, attitudes and experience.^2,3^ Little is known about these aspects, although they have a drastic influence on treatment and outcome of patients.^4^ In general and mental healthcare, coercion takes many forms, ranging from ‘formal coercion’ including involuntary admission, actions restricting movement (seclusion, restraint) and forced medication, to ‘informal coercion’ including use of influence, pressure and manipulation.^5,6^ Coercive measures infringe on fundamental human rights, and clinicians are required by law to limit freedom restricting measures in case of acute endangerment to self or others to an inevitable minimum.^7,8^ Nonetheless, until today, the use of coercion is inextricably linked to society’s images of psychiatric practice.^9^ In light of this, the World Psychiatric Association (WPA) has recently emphasised the urgency of developing and implementing evidence-based alternatives to coercive measures, calling on mental health professionals and institutions worldwide to support a rights-based, recovery-oriented approach to care.^10^ In parallel, the World Health Organization (WHO) and WPA have highlighted the need for coordinated efforts to improve quality of care and uphold human rights, with the WHO aiming to eliminate coercion altogether and the WPA focusing on the implementation of alternatives to coercion.^10,11^ Initiatives from psychiatric institutions and civil society to address problematic practices and ameliorate systems of mental healthcare are manifold.^12^ A considerate culmination point for change was the ratification of the CRPD, especially the formulation of its Articles 12 (‘Equal recognition before the law’), 14 (‘Liberty and security of person’) and 15 (‘Freedom from torture or cruel, inhuman or degrading treatment or punishment’).^1^

Use of coercive measures is known to be heterogeneously distributed among different countries because of structural, legal and cultural preconditions, but also, within countries there are substantial variations in the use of coercion between institutions.^2,13–17^ Previous studies identified influencing factors and distinguished patient-, institution- and staff-specific factors increasing or decreasing the risk for the use of coercion.^2,18^ Attitudes to coercion of psychiatric staff might explain the variations to some extent, but evidence is sparse.^3,18–20^ In the past 15 years, staff attitudes have been measured using the Staff Attitude to Coercion Scale (SACS) most frequently.^21^ SACS is the only standardised and repeatedly validated questionnaire, and has predominantly been applied in studies evaluating variation of coercion and interventions aimed at reducing coercive measures.^22^ In these studies, nurses were on average more positive toward coercive measures than psychiatrists and psychologists,^3,4,23^ but nurses’ attitudes seem to have shifted from the paradigm ‘coercion as therapeutic’ to ‘coercion as safety’ in the past decades.^24^ Differences between genders were only inconsistently found in some studies.^4,21,25^ Authors of one study claimed that staff over the age of 40 years might see coercion as more offending than younger individuals, but confirming results are missing.^4,21,22^ Concomitantly, longer work experience was correlated to a more critical view of coercion in some studies, but effects were not controlled for other influencing factors.^4,26,27^ In summary, there are several publications on staff attitudes with small to medium sample sizes, and from single centres or regions. No study has reported attitudes to coercive measures in a sample representative for staff on a national or supraregional level. This limits generalisability to larger contexts. Apart from that, previous research targeted staff at adult psychiatric institutions, and there are no data from forensic psychiatric settings or psychiatric staff working with underage patients in child and adolescent psychiatric departments. Most studies recruited mainly physicians and nurses who are most frequently involved in the use of coercion.^22,23,25,28^ Nonetheless, this could underestimate the influence of multiprofessional team dynamics that are relevant for decision-making processes.

The main objective of this study was to investigate attitudes toward coercive measures in a large and diversified sample of all mental health professionals. Special focus was placed on assessing differences in attitudes based on professional background and psychiatric setting (adult, child and adolescent, and forensic psychiatry). A secondary aim was to confirm or refute previously described differences in attitudes by gender, age, work experience and professional affiliation.

## Method

### Study design and setting

As part of the nationwide Attitudes toward Coercion project, a cross-sectional study was conducted that included psychiatric staff in an anonymous online survey across all federal states in Germany from 31 March to 7 June 2023. Direct invitations to the heads of all German psychiatric departments were sent out repeatedly, asking for dissemination of the invitation email including a common entry link to their employees. Apart from that, the study was promoted via newsletter of the German Association of Child and Adolescent Psychiatry, and via an Association of Psychiatric Clinic Directors at General Hospitals. The online survey was generated with SoSci Survey (version 3.5.02 for iOS; SoSci Survey GmbH, Munich, Germany; https://www.soscisurvey.de).

### Participants and ethics

All mental health professionals over the age of 18 years, with patient contact and working in adult psychiatry, forensic psychiatry or child and adolescent psychiatry, including in-patient and out-patient units were eligible to participate. Bias owing to socially desired answering was limited by using an anonymous survey. We aimed to generate a broad and diversified sample of psychiatric staff of all professions working in Germany. Written informed consent was obtained from all study participants. The Ethics Committee of the Charité University Medicine Berlin delivered a positive vote (approval number EA4/197/22). The authors assert that all procedures contributing to this work comply with the ethical standards of the relevant national and institutional committees on human experimentation and with the Helsinki Declaration of 1975, as revised in 2013. Study reporting followed the Strengthening the Reporting of Observational Studies in Epidemiology (STROBE) guidelines.^29^

### German legislation on coercive measures

Use of any freedom-restricting device like belts, bedrails or restricting blankets is to be recorded as mechanical restraint. Physical restraint (holding) is rare in Germany. Seclusion is defined as locking a person in a room designed for this purpose (low risk of self-harm). Chemical restraint is uncommon, forced medication is administered only in case of acute endangerment or after a court’s decision. Any coercive measure is legally based on either one of the (16 different) state laws (Psychisch-Kranken-Gesetz), issued by psychiatrists at psychosocial services in the case of acute endangerment of self or others, or on a federal law (German Civil Code, Bürgerliches Gesetzbuch) in the case of endangerment of self in patients for whom a legal guardian applies for coercive treatment.

### Measures/variables

#### Sociodemographic characteristics

Participants’ age in years, gender, professional group affiliation (e.g. ‘nurse’), workplace setting (in-patient, day hospital, out-patient), specialisation (e.g. ‘adult psychiatry’), work experience in years and federal state (optional) were assessed.

#### Staff Attitude to Coercion Scale

Data on staff attitudes regarding the use of coercion were collected using the validated German version of the SACS.^28^ The SACS consists of 15 statements on coercion, and was originally divided into three subscales:^21^ coercion as offending (critical attitude), coercion as care and security (pragmatic attitude) and coercion as treatment (positive attitude). Participants were asked to rate how strongly they agree or disagree with a particular statement on a five-point Likert scale ranging from ‘disagree strongly’ (1) to ‘agree strongly’ (5). The score for each subscale is calculated by summing up the corresponding items from each subscale.

Alternatively, several studies, mainly in German-speaking countries, did not maintain the three-factor structure, but switched to a one factor solution on a pro-con coercion spectrum.^28^ In this tradition, a mean total SACS score was calculated by reversing the items in the ‘coercion as offending’ scale and finally forming a mean of all 15 items. A higher total mean score (maximum 5) indicates a more positive attitude toward coercion. To assess which factor structure fits the data in this study better, we performed an exploratory factor analysis: Kaiser–Meyer–Olkin criterion was 0.87; and cut-off for factor loadings set to 0.3; varimax rotation was used. Anti image correlation showed values >0.75 for all items, and Eigenvalue >1 indicated a best fit for a three-factor structure. However, items loaded on multiple factors, and after adapting for a one-factor solution, all items loaded on this factor either strongly positively or negatively (the nine items from ‘coercion as care and security’ and ‘coercion as treatment’ loaded positively, the six items from ‘coercion as offending’ negatively). Therefore, and in accordance with Efkemann et al,^28^ we decided to maintain the one-factor solution for our analysis.

### Statistical methods

All statistical analyses were performed with SPSS Statistics (version 29 for iOS, IBM, Armonk, NY, USA; https://www.ibm.com/products/spss-statistics). Level of significance was set at *P* < 0.05. Descriptive statistics were used to describe participants’ sociodemographic and professional characteristics, using number and percentage for categorical variables, and mean and s.d. for continuous variables, as well as their attitudes toward coercion (mean, s.d.).

Differences in SACS mean scores between representative groups were assessed by calculating unifactorial variance analyses (analysis of variance). Groups with small sample sizes or limited patient contact, including medical assistance professions, administrative staff, staff in training and those categorised as not otherwise specified/unknown, were excluded from the inferential analyses. To correct for the influence of gender, age and work experience, multiple linear regression was used to assess the associations between profession, specialisation and attitudes to coercion.

## Results

### Participants

**Fig. 1:**
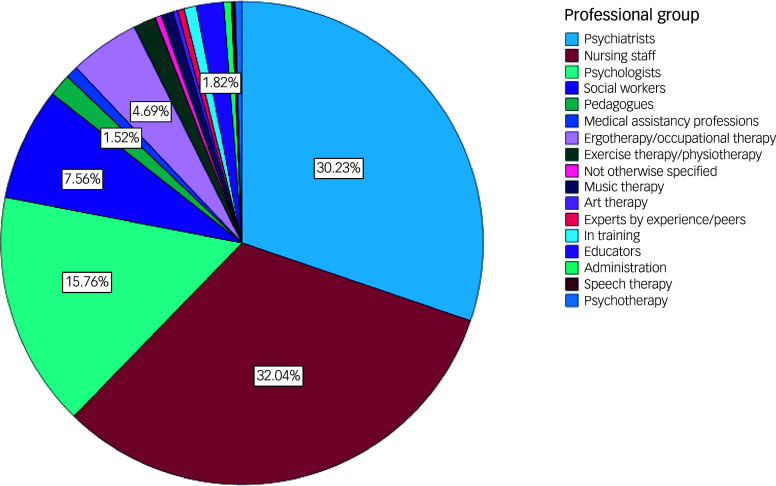
Distribution of professions.

In total, 1702 individuals from all 16 German federal states completed the online questionnaire. Mean age was 42.1 years (s.d. = 11.8 years), and mean work experience was 13.3 years (s.d. = 10.9 years). The whole sample was predominantly female (nearly two-thirds), and most participants were working in adult psychiatry, and in in-patient settings. Characteristics of all participants are given in Table 1, and distribution of professions in Fig. 1.

**Table 1:** Characteristics of participants (*N* = 1702)

Variable	*n* (%)
Gender	
Diverse	7 (0.4)
Female	1105 (64.9)
Male	590 (34.7)
Age	
18 to 25 years	93 (5.5)
26 to 35 years	535 (31.4)
36 to 45 years	417 (24.5)
46 to 55 years	342 (20.1)
56 to 70 years	315 (18.5)
Specialisation	
Adult psychiatry	1090 (64.0)
General psychiatry	941 (55.3)
Geriatric psychiatry	91 (5.3)
Psychosomatics	28 (1.6)
Psychiatry of intellectual disability	21 (1.2)
Emergency unit	9 (0.5)
Forensic psychiatry	254 (14.9)
Child and adolescent psychiatry	358 (21.0)
Work experience	
<6 years	572 (33.6)
6 to 10 years	306 (18.0)
11 to 20 years	375 (22.0)
21 to 30 years	307 (18.0)
31 to 40 years	130 (7.6)
>40 years	12 (0.7)
Workplace setting	
In-patient	1344 (79.0)
Out-patient	228 (13.4)
Day hospital	130 (7.6)

### Staff attitudes to coercion

The count of valid SACS questionnaires was 1702. Out of the three original subscales, ‘coercion as care and security’ (pragmatic attitude) received strongest agreement, with an overall mean score of 3.78 (s.d. = 0.59). Item 2 (‘For security reasons, coercion must sometimes be used’) was the item with the highest mean score (mean 4.10, s.d. = 0.66) belonging to this subscale. However, the single item with the highest agreement (mean 4.20, s.d. = 0.91) was item 15 (‘Coercion could have been much reduced, giving more time and personal contact’) from the subscale ‘coercion as offending’ (mean 3.57, s.d. = 0.65). The items on the subscale ‘coercion as treatment’ had the lowest agreement, on average (mean 2.14, s.d. = 0.75). The detailed results of all SACS items are listed in Table 2.

**Table 2:** Staff Attitude to Coercion Scale mean overall score and item means (*N* = 1702)

Item	Item	Mean (range 1–5)	s.d.
1–15	**Mean overall score**	**2.91**	**0.51**
1	Use of coercion is necessary as protection in dangerous situations	3.96	0.79
2	For security reasons, coercion must sometimes be used	4.10	0.66
3^a^	Use of coercion can harm the therapeutic relationship	4.08	0.86
4^a^	Use of coercion is a declaration of failure on the part of the mental health services	2.37	1.02
5	Coercion may represent care and protection	3.74	0.83
6	More coercion should be used in treatment	1.80	0.91
7	Coercion may prevent the development of a dangerous situation	3.49	1.02
8^a^	Coercion violates the patient’s integrity	3.82	0.90
9	For severely ill patients, coercion may represent safety	3.70	0.87
10	Patients without insight require use of coercion	2.38	1.00
11	Use of coercion is necessary toward dangerous and aggressive patients	3.71	0.95
12	Regressive patients require use of coercion	2.23	0.87
13^a^	Too much coercion is used in treatment	2.97	1.01
14^a^	Scarce resources lead to more use of coercion	3.98	1.01
15^a^	Coercion could have been much reduced, giving more time and personal contact	4.20	0.91

a. These items were reversed (*x* = 6−*y*) for building mean overall score.

### Professional group- and specialisation-defined attitudes to coercion

SACS mean overall scores are listed according to professional group and specialisation in Table 3. Experts by experience were most critical towards the use of coercion in this study (mean 2.39, s.d. = 0.57). Groups with highest SACS overall attitude scores were medical assistance professions (mean 3.17, s.d. = 0.33), people in training (mean 3.15, s.d. = 0.42) and administrative personnel (mean 3.12, s.d. = 0.24), as well as staff in the youngest age group under 26 years (mean 3.06, s.d. = 0.49). Staff in adult psychiatric settings showed varying levels of overall SACS attitude scores, with less critical attitudes observed in geriatric psychiatry (mean 3.01, s.d. = 0.50), emergency psychiatry (mean 3.00, s.d. = 0.57) and psychosomatics (mean 2.96, s.d. = 0.58), compared with settings for people with intellectual disabilities (mean 2.75, s.d. = 0.49) and general psychiatry (mean 2.88, s.d. = 0.54).

**Table 3:** Staff Attitude to Coercion Scale (SACS) mean scores comparing gender, age, work experience, professions and specialisations (*N* = 1702)

	SACS overall attitude (mean (s.d.), 1–5)
Gender (*n*)	
Diverse (7)	2.85 (0.87)
Female (1105)	2.90 (0.51)
Male (590)	2.93 (0.52)
Age interval (*n*)	
18 to 25 years (93)	3.06 (0.49)
26 to 35 years (595)	2.87 (0.51)
36 to 45 years (417)	2.94 (0.51)
46 to 55 years (342)	2.93 (0.50)
56 to 70 years (315)	2.88 (0.52)
Work experience (*n*)	
<6 years (572)	2.92 (0.49)
6 to 10 years (306)	2.89 (0.52)
11 to 20 years (375)	2.89 (0.54)
21 to 30 years (307)	2.94 (0.52)
31 to 40 years (130)	2.87 (0.53)
>40 years (12)	2.93 (0.40)
Professional group (*n*)	
Psychiatrists (515)	2.89 (0.46)
Nursing staff (545)	3.00 (0.55)
Psychologists (267)	2.77 (0.50)
Social workers (128)	2.91 (0.58)
Pedagogues (26)	2.89 (0.42)
Medical assistance professions (14)	3.17 (0.33)
Ergotherapy/occupational therapy (80)	2.85 (0.47)
Exercise therapy/physiotherapy (26)	2.96 (0.46)
Not otherwise specified/unknown (8)	3.28 (0.37)
Music therapy (14)	2.71 (0.53)
Art therapy (5)	2.51 (0.80)
Experts by experience/peers (8)	2.39 (0.57)
In training (14)	3.15 (0.42)
Educators (31)	3.01 (0.30)
Administration (9)	3.12 (0.24)
Speech therapy (4)	2.75 (0.27)
Psychotherapy (8)	2.57 (0.59)
Specialisation	
Adult psychiatry (1090)	2.89 (0.53)
General psychiatry (941)	2.88 (0.54)
Geriatric psychiatry (91)	3.01 (0.50)
Psychosomatics (28)	2.96 (0.58)
Psychiatry of intellectual disability (21)	2.75 (0.49)
Emergency unit (9)	3.00 (0.57)
Forensic psychiatry (254)	3.01 (0.50)
Child and adolescent psychiatry (358)	2.91 (0.44)

### Effects of sociodemographic and professional characteristics on SACS outcome

Using analysis of variance, there were no significant differences between male, female and diverse participants in SACS mean scores (*F*(2) = 1.07, *P* = 0.342). Regarding age, there was a significant effect of age group on SACS score (*F*(4) = 3.79, *P* = 0.005), with the group between 18 and 25 years showing the least critical attitudes. Work experience had no significant effect (*F*(5) = 0.47, *P* = 0.798). Specialisation in adult, forensic or child and adolescent psychiatry had a significant effect on SACS score (*F*(2) = 5.793, *P* = 0.003), with staff in forensic psychiatry overall showing less critical attitudes than staff in the other two specialisations. Finally, there was a significant effect of professional group affiliation on SACS mean score (*F*(12) = 4.75, *P* < 0.001); mean SACS scores for all subgroups are given in Table 3.

However, in multiple regression analysis, specialisation and profession remained significantly associated with SACS mean score, when controlling for gender, age and work experience. Output of multiple regression is given in Table 4.

**Table 4:** Multiple regression analysis with Staff Attitude to Coercion Scale mean score as dependent variable

Variables	*B*	*β*	s.e.
Constant	2.901 (*P* < 0.001)*		0.078
Gender	−0.032 (*P* = 0.226)	−0.030	0.026
Age	0.000 (*P* = 0.889)	0.006	0.002
Work experience	−0.001 (*P* = 0.714)	−0.015	0.002
Professional group affiliation	−0.013 (*P* = 0.008)*	−0.067	0.005
Specialisation	0.060 (*P* < 0.001)*	0.087	0.017
Observations	1657		
*R*^2^	0.011		
Adjusted *R*^2^	0.008		
Residual s.e.	0.513		
*F* statistics (d.f. = 5)	3.826 (*P* = 0.002)		
Highest VIF	2.744 (work experience)		

VIF, variance inflation factor.

*P < 0.05.

## Discussion

### Key results

This study succeeded in reaching a large and diversified sample of psychiatric staff of all professions on a national level and is by far the largest study on attitudes toward coercion worldwide. In the context of other research studies using the SACS, there is low approval of coercion as treatment,^20,30^ whereas it is frequently seen as threatening the therapeutic relationship and offending patient rights and integrity.^18^

Staff in forensic psychiatric settings had less critical views on coercion when compared with staff from adult psychiatric and child and adolescent psychiatric departments. Overall attitude to coercion was not different by gender. Bivariate differences in attitude because of age and work experience did not persist when correcting for the influence of specialisation and profession. Members of different professions in multidisciplinary psychiatric staff showed small to medium, but significant, differences in approaches to coercion dependent on their professional affiliation. Compared with physicians, nurses were less critical in this study, whereas psychologists and therapeutic professionals were more critical. Experts by experience were the most critical subgroup.

### Interpretation

In conjunction with the evidence from published studies worldwide,^22^ and especially compared with the most recent German study from 2020,^28^ approval of coercion in our sample was relatively low. Only one single-centre study in Germany, conducted at a hospital with recovery-oriented psychiatric care concepts, reported even more critical views.^3^ Coercion in psychiatry is predominantly perceived as a necessary tool for the prevention of acute endangerment. At the same time, there are signs of increased recognition of hazards of coercive measures and a disapprobatory attitude toward coercion. Overall, therapeutic or positive effects are decreasingly attributed to the use of coercion, which can be seen as an effect of the increasingly critical debate on coercion in psychiatry, associated with the introduction of the CRPD^1^ and S3 guideline recommendations from the national association.^31^ Recent policy statements by the WPA and WHO highlight the international shift toward rights-based, recovery-oriented care, and call for reducing or eliminating coercive practices in mental health services.^10,11^

On the individual level, differences in attitudes toward coercion are only partially explained by the variables assessed in this study, being gender, age, work experience, profession and specialisation (adjusted *R*^2^ = 0.008). Nonetheless, there are consistent differences in attitudes between professions. One conjecture is that individuals in professions that are obliged to use coercion more frequently show a less critical attitude. This would explain the less critical views on coercion held by psychiatrists and nurses; however, other groups like medical assistance professions or administrative personnel were even less critical. Another presumption is that attitudes depend on the degree of contact with patients, and that there is an association between longer and closer relationships to their clients and more critical attitudes toward the use of coercive measures. Perhaps it is not the amount of time spent with the patient, but rather the quality of the therapeutic relationship, that might mitigate this effect. Coercion-critical attitudes of experts by experience, as well as of professions with therapeutic backgrounds, would sustain this explanation. Nurses are often the primary contact persons for people in psychiatric hospitals, but are often responsible for creating structure and safety for a group of patients. More importantly, nurses are most frequently confronted with violence and might be less critical toward coercion because of structural deficits like staff shortage, overcrowding, safety concerns and own symptoms of stress.

In conclusion, differences between professions seem less pronounced in relation to the common efforts of multidisciplinary teams in psychiatric departments to reduce the use of coercion. Underlying causal factors for differences in attitudes, yet unknown, might inform future strategies for reducing coercion in psychiatry. Based on this study, we think that advocacy is needed to optimise structural factors and to implement ethical reflections of attitudes and emphasise rights-based initiatives in the training of psychiatric staff.

Staff in forensic psychiatric institutions are faced with legal and medical challenges in the care for the patients who are admitted on involuntary terms and for longer periods of time. In forensic psychiatric practice, professionals must emphasise safeguarding of patient and ward, risk management and control orientation in their day-to-day work.^32^ Recovery-oriented practices are a young and evolving field, and so far limited to singular initiatives, like the adaptation of the ‘Good Life Model’ in New Zealand.^33^ In this context, the moderately, but significantly less critical, attitude in forensic psychiatric staff toward coercion can be interpreted in the context of their workplace being characterised by involuntary treatment and constant risk assessment. The observed difference warrants further investigation of the attitudes of staff working in forensic psychiatric settings in future studies.

### Limitations

This study has some limitations. First, explicit attitudes were investigated, and no measure was used for implicit attitudes. Respondents might have been influenced by social desirability. The SACS questionnaire does not give contextual information, and there is no differentiation between different types of coercive measures like restraint or seclusion. Therefore, associated topics and situations might differ between individuals. More distinguished results could be achieved by asking for staff attitudes in defined situations such as case vignettes as well as using qualitative study designs. Second, motivation for participation in the study was possibly higher if people were already interested in the topic of coercive measures, resulting in potential selection bias. Finally, although a large sample size is helpful, the inclusion of various specialisations, multiple hospitals and different structural surroundings may have added heterogeneity and diversity to the study sample, and some subsamples were small.

### Generalisability

These results provide the opportunity to investigate approaches toward coercion in psychiatric practice, with its large scale and nationwide study sample. Staff from child and adolescent psychiatric departments, forensic psychiatric institutions and different adult psychiatric settings, as well as multitudes of different professions, were included in the sample. Therefore, we were able to assess attitudes across various subgroups. The use of SACS makes the results comparable to recent research in this area. However, we are still not sure how staff attitudes are linked to the actual use of coercion. Nonetheless, this research raises important questions that need be answered to shed light on the complex interplay between attitudes and the interaction between psychiatric professionals and their patients.

In conclusion, staff attitudes toward coercion are evolving and changing because of sociocultural and legal changes. Our data from Germany reveal both pragmatic and critical attitudes toward coercive measures, with an emphasis on patient rights and the protection of individuals’ integrity. Coercion is generally not seen as therapeutic. Differences in attitudes are mainly linked to professional training and structural surroundings like specialisation of psychiatric institution (forensic psychiatric versus general psychiatric and child and adolescent psychiatric). Further studies are warranted to differentiate how mental health professionals experience coercion, and how the actual use of coercion is influenced by staff attitudes.

## Data Availability

The data that support the findings of this study are available from the corresponding author, J.S.B., upon reasonable request.
